# Factors Affecting Engagement in Web-Based Health Care Patient Information: Narrative Review of the Literature

**DOI:** 10.2196/19896

**Published:** 2021-09-23

**Authors:** Liam Alperen Oktay, Eyad Abuelgasim, Aida Abdelwahed, Nour Houbby, Smaragda Lampridou, Pasha Normahani, Nicholas Peters, Usman Jaffer

**Affiliations:** 1 Imperial College London London United Kingdom; 2 Imperial College NHS Trust London United Kingdom

**Keywords:** patient education, web-based health information, internet, patient engagement, mobile phone

## Abstract

**Background:**

Web-based content is rapidly becoming the primary source of health care information. There is a pressing need for web-based health care content to not only be accurate but also be engaging. Improved engagement of people with web-based health care content has the potential to inform as well as influence behavioral change to enable people to make better health care choices. The factors associated with better engagement with web-based health care content have previously not been considered.

**Objective:**

The aims of this study are to identify the factors that affect engagement with web-based health care content and develop a framework to be considered when creating such content.

**Methods:**

A comprehensive search of the PubMed and MEDLINE database was performed from January 1, 1946, to January 5, 2020. The reference lists of all included studies were also searched. The Medical Subject Headings database was used to derive the following keywords: “patient information,” “online,” “internet,” “web,” and “content.” All studies in English pertaining to the factors affecting engagement in web-based health care patient information were included. No restrictions were set on the study type. Analysis of the themes arising from the results was performed using inductive content analysis.

**Results:**

The search yielded 814 articles, of which 56 (6.9%) met our inclusion criteria. The studies ranged from observational and noncontrolled studies to quasi-experimental studies. Overall, there was significant heterogeneity in the types of interventions and outcome assessments, which made quantitative assessment difficult. Consensus among all authors of this study resulted in six categories that formed the basis of a framework to assess the factors affecting engagement in web-based health care content: easy to understand, support, adaptability, accessibility, visuals and content, and credibility and completeness.

**Conclusions:**

There is a paucity of high-quality data relating to the factors that improve the quality of engagement with web-based health care content. Our framework summarizes the reported studies, which may be useful to health care content creators. An evaluation of the utility of web-based content to engage users is of significant importance and may be accessible through tools such as the Net Promoter score. Web 3.0 technology and development of the field of psychographics for health care offer further potential for development. Future work may also involve improvement of the framework through a co-design process.

## Introduction

### Background

In the United Kingdom, up to two-third use the internet to obtain health-related information at some point in their journey [1,2]. The internet has become an important source of education for patients, who are increasingly expected to, and are motivated to, play an active role in making decisions related to their health [3]. Patient education is defined as “the process by which health professionals and others impart information to patients that will alter their health behaviours or improve their health status” [4]. This may include information that is factual or related to patient experience, depending on the issue being addressed [5,6]. Reports suggest that 70% of the patients would like their physicians to recommend a source of web-based information relating to their medical condition, but only 4% of the patients receive such a recommendation [7].

Web-based patient health care information has several potential benefits, including convenient 24-hour access potentially wherever you are, ability to enhance knowledge acquisition [8,9], reduce anxiety [9], and improve the quality of conversations during health-related encounters. However, the effect of web-based content on patient empowerment, self-efficacy, and health attitudes has been found to be variable [9]. This may be due to the absence of an evidence-based framework outlining the factors that should be considered for improving engagement with web-based health care information.

### Objectives

In this narrative review, we aim to identify and evaluate the factors that should be considered when producing engaging and high-quality web-based health care patient information. We also aim to incorporate these findings into a framework that may be useful as a guide to developing web-based health care information.

## Methods

### Overview

A comprehensive search of the PubMed and MEDLINE database was performed from January 1, 1946, to January 5, 2020. The Medical Subject Headings database was used to derive keywords and search term combinations, which included “patient information,” “online,” “internet,” “web,” and “content.” All studies pertaining to the factors affecting engagement in web-based health care patient information were included. No restrictions were set on the study type. Only studies in English were included. Analysis of themes arising from the results was performed using inductive content analysis. All retrieved abstracts and titles were reviewed by 2 independent investigators (EA and LAO) for relevance pertaining to engagement with web-based health care content. Disagreements between the reviewers were solved by consensus. Manual cross-checking of the reference lists of the identified papers was carried out to identify any other potentially relevant studies.

Analysis of themes arising from the results was performed using inductive content analysis [10]. This involved the reviewing of titles and abstracts by an author (UJ), with free generation of the categories relating to *factors associated with high-quality and engaging web-based content*. The categories were named using content characteristic words, and these were expanded into subcategories. The number of times a category was reported in the articles was totaled, and a list of categories was created in order of the frequency of mentions. All authors reviewed this list to decide which categories would be included in the final list.

### Eligibility Criteria

As this is the first narrative review of its kind, no limitation was placed on study type or on surrogate measures of the outcome described.

### Outcomes

All studies relevant to quality of engagement in web-based health care content were included.

## Results

### Overview

**Figure 1:**
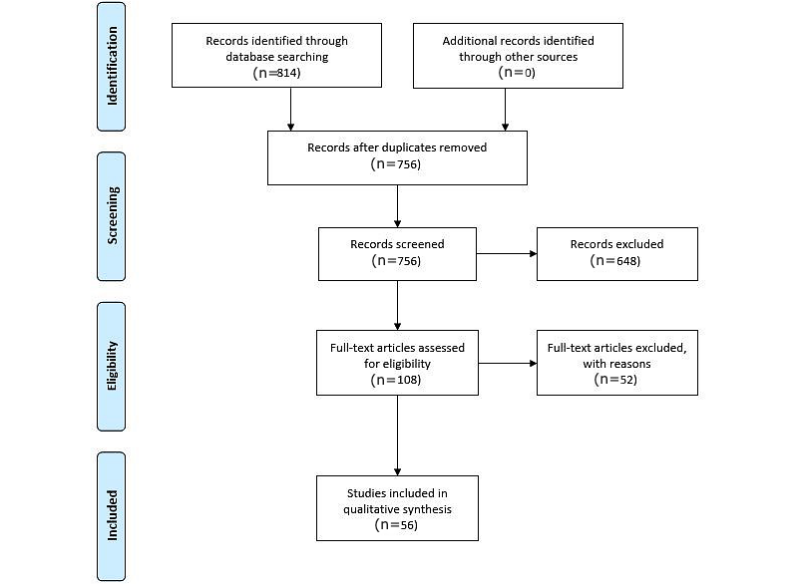
PRISMA (Preferred Reporting Items for Systematic Reviews and Meta-Analyses) flowchart showing article selection.

A total of 814 articles were identified, and of these, 108 (13.3%) were selected for full-text review based on their title and abstract. Full-text screening of the 108 articles resulted in the final selection of 56 (51.9%) articles, from which seven categories were derived. Figure 1 shows the Preferred Reporting Items for Systematic Reviews and Meta-Analyses flowchart which depicts the stages of article selection.

### Description of Studies

#### Development of Categories

The studies were mostly observational and qualitative. A total of 3 randomized controlled trials and 5 systematic reviews were also identified. Categories relating to *factors associated with high-quality and engaging web-based content* were developed according to themes that were found to arise in the studies identified. The categories settled upon were as follows (with the number of papers reporting included in parentheses): (1) textual information (16 papers); (2) discussion boards or web-based groups (3 papers); (3) video content (11 papers); (4) visuals or pictographs (1 paper); (5) device accessibility (12 papers); (6) stage of patient journey (8 papers); and (7) credibility and completeness of information (4 papers; Table 1).

**Table 1:** Studies pertaining to engagement with web-based content. Studies are according to mode of engagement (N=56).

Study			Study type			Content		Outcomes pertaining to quality of web-based health care patient content		Main results
**Textual information**										
	Chedid et al (2018) [11]	Observational study			Government-hosted website, textual, and visual aids for prenatal health promotion		Comprehensiveness, evidence-based information, accessibility, and inclusivity. Minimum of three referenced prenatal health topics necessary to be classed as evidence-based.		Assessment of prenatal health promotion material revealed that 66.4% of the federal, 84.6% of the provincial or territorial, and 80% of the public health regional unit–hosted websites, and 87.5% of the e-classes were evidence based. Only 25% of the municipal websites met this standard. No *P* values stated.	
	Ernst et al (2019) [12]	Observational study			Disorders of sex development, affiliated health care system’s web-based information		The SMOGa Readability Formula determined reading level, the PEMATb evaluated content for understandability and actionability, and the DISCERN Tool assessed treatment decision-making information.		Reading level of webpages as determined with the SMOG Readability Formula met or exceeded high school grade level. The mean PEMAT understandability score for team pages and team links was 68% (SD 6%). On average, the pages met less than 70% of the understandability criteria. The mean PEMAT actionability score was 23% (SD 20%). The DISCERN Tool found that the quality of information relating to hormone treatment and to surgery was poor. No *P* values stated.	
	Hjelmager et al (2019) [13]	Qualitative study			Web-based information material for patients with low back pain in general practice discussed in the context of app development		Barriers to and facilitators for future use of the health information technology app for patients with low back pain.		Eight 1-hour interviews with general practitioners revealed the following: content for lower back pain should be validated by general practitioners; from a trustworthy source; support ongoing treatment plan. No *P* values stated.	
	Rofaiel et al (2018) [14]	Observational study			Websites that describe the biologic agents used as treatment options for inflammatory bowel disease		The DISCERN model was used to evaluate the quality of the information content.		The mean DISCERN score across all websites was 3.21 out of a 5-point scale. No significant difference was found between patient-searched and physician-recommended websites, with a mean score of 3.21 versus 3.63, respectively (*P*=.16).	
	Alfonso et al (2019) [15]	Observational study			American Cleft Palate–Craniofacial Association–approved teams’ websites		Content and readability of team websites.		The mean reading level 10.7 (SD 1.9) exceeded the American Medical Association-recommended sixth grade reading level. Children’s Hospital–affiliated teams (n=86) yielded significantly higher content scores (14.8 vs 13.5; *P*=.03). Children’s Hospital teams also had better readability as indicated by lower reading grade level (10.5 vs 11.4; *P*=.04).	
	Ayyaswami et al (2019) [16]	Observational study			Web-based cardiovascular disease–related health education articles accessed through Google		Readability according to 10 readability measures (Flesch Reading Ease, Coleman-Liau Index, Flesch-Kincaid Grade Level, Gunning Fog Index, FORCAST Readability Formula, New Dale-Chall formula, New Fog Count, SMOG Index, Fry Readability Formula, and Raygor Readability Estimate).		All measures that assessed mean reading grade level found that 196 articles were written at a mean 10.9 (SD 1.8) grade reading level. 99.5% of the articles were written beyond the fifth to sixth grade reading level. No *P* values stated.	
	Fajardo et al (2019) [17]	Systematic review			Web-based deprescribing patient education materials		PEMAT and International Patient Decision Aids Standards Inventory. Readability using Gunning Fog Index and Flesch-Kincaid Grade Level.		Patient education materials addressing deprescribing of medications for symptom control (81%) were most common. 37% of deprescribing patient education materials present potential benefits and harms of deprescribing. Most patient education materials are pitched above average reading levels (average minimum reading level of grade 12). No *P* values stated.	
	Vivekanantham et al (2017) [18]	Observational study			Web-based health information for patients with polymyalgia rheumatica		Readability using the Flesch Reading Ease and SMOG Readability Formula tools. 8-item Credibility Indicator (incorporating authorship, affiliation, editorial team, date of creation, date of update, backing, accreditation, and financing).		Of the websites identified (n=52), the mean Flesch Reading Ease and SMOG Readability Formula scores were 48 (SD 15) and 10 (SD 2), respectively. The mean Credibility Indicator was 2 (SD 1). Of 52 websites, 50 (96%) of the websites were accurate. No *P* values stated.	
	Harris et al (2018) [19]	Cross-sectional descriptive			Evaluation of leading web-based content on tympanostomy tube placement		PEMAT understandability and actionability scores		The PEMAT scores found that all sites (n=10) were understandable (mean 81.9%). Seven of the sites had a low actionability score (mean 44.6%). No *P* values stated.	
	Maciolek et al (2017) [20]	Observational study			Prostate biopsy web-based patient education materials		Readability was assessed using Flesch-Kincaid Grade Level. Understandability and actionability were measured using the PEMAT.		Of the 148 sites, 31 (20.9%) met the recommended below eighth grade reading level. The PEMAT understandability score for academic institution–sourced patient education materials was higher than that for patient education materials sourced from the private sector (*P*=.02) and from institutions unaffiliated with urologists (*P*=.01).	
	Siddhanamatha et al (2017) [21]	Observational study			Websites providing educational content for patients with rheumatoid arthritis		Accuracy, completeness, technical elements, design and aesthetics, readability, usability, and accessibility of the websites		Of 46 websites in total, 45 (98%) provided accurate information. The mean reading level was grade 12.1 (SD 2.3). In total, 78% (36/46) were easy to navigate, but only 33% (15/46) were user friendly for people with visual and or hearing impairments. No *P* values stated.	
	Nielsen-Bohlman et al (2004) [22]	Observational study			An evaluation of health literacy in the United States; formulate solution to overcome associated obstacles		Current level of readability of web-based content		More than 300 studies indicate that health-related materials exceed the average reading grade level of US adults. No *P* values stated.	
	Chin et al (2018) [23]	Observational study			Improve patient understanding of web-based content pertaining to adults with hypertension.		Information retention and comprehension		The revised passages yielded improved retention and comprehension, with less reading time required per unit uptake of information also noted. The methods included simplifying language and signaling clear organization. On average, the participants were found to significantly better remember the revised passages (mean 0.74, SD 0.14) compared with the typical passages (mean 0.70, SD 0.11; *P*<.01).	
	Boudewyns et al (2015) [24]	Randomized controlled trial			Web-based information handouts		Information comprehension and application		Individuals who received the revised and improved web-based formats had higher comprehension scores than those who received the MedGuide (*P*<.001).	
	Morrow et al (2005) [25]	Observational study			Patient-centered medication instructions to empower patients to plan a medication-taking regimen		Information comprehension and retention, health-related literacy, and verbal working memory		Patient-centered instructions were more accurately understood for unfamiliar medications (*P*<.05). The standard instructions were more accurate for familiar medications (*P*<.05).	
	Pander Maat et al (2010) [26]	Observational study			Revision of patient information leaflets		Usability, speed of information retrieval, and comprehension.		Once located, comprehension of the information was approximately 90%. Revisions led to better performance. Information was found more quickly. Comprehension scores were also improved. No *P* values stated.	
**Discussion boards or web-based groups**										
	Cedars et al (2019) [27]	Qualitative and thematic analysis			Web-based discussion boards for urethral stricture disease and urethroplasty		To describe the patient experience and chief concerns with urethroplasty to improve physician understanding and patient education To understand how men use web-based discussion boards and what information is available about urethroplasty		Problems in navigating the health care system with urethral stricture disease (n=141) and weak urine stream (n=70) were the most frequent preurethroplasty complaints. The patients participated in web-based discussions to share experiences with urethral stricture disease and urethroplasty, share emotional support, and search for answers. No *P* values stated.	
	Teaford et al (2019) [28]	Descriptive statistics and qualitative content analysis			New mothers’ experiences with web-based postpartum forums		To explore women’s experiences with a web-based forum during the postpartum period		Five themes were produced through data analysis: (1) social support, (2) anonymity, (3) in-groups, (4) drama, and (5) entertainment or pastime. The participants found that the discussion board could facilitate sharing of information, act as an entertainment source, and provide community. No *P* values stated.	
	Castaneda et al (2019) [29]	Qualitative study			eHealth peripheral artery disease community forums		Original posts and related responses were analyzed for thematic content.		The themes identified included medical advice (41%), personal experiences with peripheral artery disease (33%), and social support (13%). Negative attitudes were discussed in 10 of the 18 (56%) posts related to poor experiences with health care providers; 15.1% of the medical advice was inconsistent with clinical treatment guidelines. No *P* values stated.	
**Video**										
	Bae et al (2018) [30]	Observational study			YouTube videos in English as a patient education resource for cataract surgery		14 criteria important for educating patients about the procedure.		The mean number of usefulness criteria satisfied was only 2.28 (SD 1.80) out of 14. There was no significant difference in view counts between the most useful videos and other videos (*P*=.94). Videos from medical bodies such as the National Health Service were found more useful in terms of patient education (*P*<.001).	
	Pedersen et al (2019) [31]	Feasibility study			Development of a preventive educational video for patients exposed to whiplash trauma		The development followed a systematic approach and was theory driven, supplemented with available empirical knowledge.		The participants (n=4) felt that the content was “relevant, helpful, and reassuring to watch.” All four preferred video content instead of written material. No *P* values stated.	
	Finnegan et al (2018) [32]	Case study			A web-based vaccine communication project (textual, videos, and infographics)		Case study of a provaccine information hub launched in 2011. Vaccines Today provides high-quality information about vaccines and diseases, expert interviews, answers to frequently asked questions, parent or patient stories, and videos or infographics.		Two categories of informing patients were found to work well: (1) the storytelling approach and (2) answering questions posed by patients. No *P* values stated.	
	Button et al (2018) [33]	Mixed methods study (qualitative and feasibility study)			A web-based intervention (TRAK^c^) that provides knee patients with health information		Testing the TRAK intervention in patients undergoing physiotherapy to gain their subjective insights into its use		The participants reported that TRAK was easy to use overall. Basic internet skills were all that were required. Using TRAK as an adjunct to physiotherapist management increased the patients’ understanding and confidence. No *P* values stated.	
	Vogel et al (2018) [34]	User survey			VaPE^d^ in anesthesia		The content of the videos, the technique of video presentation, usefulness of VaPE Interviews carried out with patients and physicians		In total, 98% (78/80) of the anesthetists found VaPE useful for patient education. In total, 93% (74/80) observed time saved for the following interview. In total, 96% (77/80) stated that watching the video left patients better informed. Increased anxiety caused by VaPE was noted by 46% (37/80); 54% (43/80) found no such effect. No *P* values stated.	
	Pithadia et al (2019) [35]	Cross-sectional study			YouTube videos as a source of patient information about phototherapy and excimer laser for psoriasis		Assess the educational quality of YouTube videos about phototherapy and excimer laser for psoriasis		In total, 11.2% (15/135) of the videos contained high-quality patient educational information, 2.5% (3/135) were fair quality, and 66.1% (89/135) were low quality. A total of 28.2% (35/135) of videos provided background information regarding psoriasis. Of these 35 videos, 28 (80%) contained evidence-based content about the epidemiology, systemic involvement, genetics, and immune nature of psoriasis. Of the 35 videos, 7 (20%) presented nonevidence-based claims and high mortality rates associated with psoriasis. No *P* values stated.	
	Ferhatoglu et al (2019) [36]	Observational study			Sleeve gastrectomy videos shared on YouTube		The popularity of the videos was evaluated with the Video Power Index. The educational quality of the videos was measured using the DISCERN score, JAMAe benchmark criteria, and GQSf. The technical quality was measured by the SGSSg.		The DISCERN, JAMA benchmark criteria, GQS, and SGSS evaluations of academic-sourced videos yielded significantly higher scores than patient-sourced videos (*P*<.001, *P*<.001, *P*=.001, and *P*<.001, respectively). However, the Video Power Index evaluation of patient-sourced videos yielded significantly higher scores than academic- and physician-sourced videos (*P*<.001 and *P*=.003, respectively). Negative correlations between the Video Power Index and the JAMA, GQS, and SGSS scores were found.	
	Erdem et al (2018) [37]	Observational study			Bariatric surgery videos (n=175) on YouTube		Usefulness of bariatric surgery videos on YouTube: A usefulness score (very useful, useful, or not useful)		Of the 175 videos, 94 (53.7%) were useful, and 43 (24.6%) were very useful. No videos were found containing misleading information. A Spearman rank correlation found no significant correlation between the usefulness score and the number of views (ρ=−0.118; *P*=.12), number of likes (ρ=−0.038; *P*=.61), number of dislikes (ρ=−0.003; *P*=.97) or video length (ρ=−0.106; *P*=.16).	
	Biggs et al (2013) [38]	Observational study			YouTube as a source of information on rhinosinusitis		Videos (n=100) were graded on their ability to inform the layperson about rhinosinusitis.		45% of the videos were deemed to provide some useful information. 55% of the videos contained little or no useful facts, 27% of which contained potentially misleading or even dangerous information. Videos uploaded by medical professionals or those from health information websites contained more useful information than those uploaded by independent users. No *P* values stated.	
	Kwok et al (2017) [39]	Observational study			Videos available on YouTube pertaining to interventional treatment for varicose veins		Informational and scientific quality (good, fair, and poor) and stance (for, neutral, against, and unclear) toward the treatment option discussed, treatment type, and video source.		The largest group of videos (47.3%) received a quality rating of fair, meaning that they discussed one or two aspects of a treatment option, such as procedural technique and indications. Among those videos rated poor (25%), nearly all (98.2%) failed to mention a specific treatment. No *P* values stated.	
	Bademci et al (2017) [40]	Observational study			YouTube videos on deep venous thrombosis		Scientific content, accuracy, and currency		Although most of the videos uploaded (25/111, 22.9%) were created by physicians, the number of views for website-based videos was significantly higher (*P*=.002). When the uploaded videos were assessed in terms of their usefulness, the videos from physicians and hospitals were statistically more useful than the other videos (*P*<.001).	
**Visuals or pictographs**										
	Christensen et al (2017) [41]	Pilot study			Doodle Health: A crowdsourcing web-based game for the co-design and testing of pictographs to reduce disparities in health care communication		To test the usability of the game and its appeal to health care consumers in the co-design and evaluation of pictographs.		Initial testing indicates that crowdsourcing is a promising approach to pictograph development and testing for relevancy and comprehension. More than 596 drawings were collected, and 1758 guesses were performed to date with 70%-90% accuracy. No *P* values stated.	
**Device on which content is accessed**										
	Gogovor et al (2017) [42]	Literature review and qualitative focus group study			Development of an internet-based chronic pain self-management program		Information needs and gaps in chronic pain management as well as technology features to inform the development of an internet-based self-management program		The gaps identified in terms of chronic pain management included lack of knowledge, limited access to health care, substandard care, and scarce self-management support. The focus group themes included patient education on chronic pain care, attitude-belief-culture, financial and legal issues, and motivational content. No *P* values stated.	
	Lüchtenberg et al (2008) [43]	Observational study			Websites containing medical information addressing laymen or patients (n=139)		Accessibility using a quantitative checklist based upon the Web Content Accessibility Guidelines of the World Wide Web Consortium		Of the 139 sites, 25 (17.9%) of the sites were categorized as WAIh guidelines level A or AA. WAI guidelines level AA was reached by 0.7% (1/139) of website. None of the websites reached level AAA. Of the 139 sites, 82% (114) of the assessed consumer websites were not completely accessible to persons who are visually impaired. No *P* values stated.	
	Bashi et al (2018) [44]	Systematic review			Patient educational interventions using mobile apps		The reporting quality of the studies was assessed according to the mHealthi evidence and predefined reporting assessment criteria.		Of the 15 studies, none reported on the data security, privacy, and confidentiality measures. No *P* values stated.	
	Noel et al (2017) [45]	Prospective cohort study			A mobile medical app was developed to improve postoperative care of patients who had undergone plastic surgery		The content, design, and efficacy of the app were assessed with a questionnaire (n=60).		The participants reported that the questions regarding postoperative management were addressed effectively, with a mean score of 4.1/5. Most of the participants recommended the app to patients who had undergone plastic surgery, with a mean score of 4.6/5. The app’s information prevented 12 patients from calling the plastic surgeon or the emergency department unnecessarily. No *P* values stated.	
	Nicholas et al (2015) [46]	Systematic review			Mobile apps for bipolar disorder		The comprehensiveness and quality of information was assessed against core psychoeducation principles and current bipolar disorder treatment guidelines. The management tools were evaluated with reference to the best practice resources for the specific area. General app features and privacy and security.		Informative apps covered more than a third (4/11, 36%) of core psychoeducation principles and 15% (2/13) of best practice guidelines. A third (10/32, 31%) cited their sources. “Neither comprehensiveness of psychoeducation information (ρ=−0.11; *P*=.80) nor adherence to best practice guidelines (ρ=−0.02; *P*=.96) were significantly correlated with average user ratings.”	
	Jamison et al (2017) [47]	Randomized controlled trial			To test an app that enables patients with chronic pain to assess, monitor, and communicate their status to their health care provider.		Frequency of app use and app satisfaction scores		In total, 78.1% (82/105) of the participants reported daily using the app. Patient satisfaction survey results: Ease of use: 1.8/10 (0=very easy to use, 10=unusable) Willingness to use after the study: 2.4/10 (0=very willing; 10=unwilling. Participants with more daily assessments reported higher app satisfaction (*P*<.05) than those who used the app less.	
	Schulz et al (2007) [48]	Randomized controlled trial			Website designed to enhance self-management in chronic lower back pain		Change in pain levels, change in knowledge, behavioral changes, and medication use		Users accessed the website an average of 11.5 times during the 5-month study. Mean pain levels fell in the control group from 5 to 3.9 (10=most severe pain imaginable, 1=no pain), whereas the mean pain levels in the control group remained largely the same (6.1 to 6.3). No *P* values stated.	
	Caiata Zufferey et al (2009) [49]	Observational study			Website *Oneself* designed to promote self-management and inform patients on lower back pain management		Self-comprehension Improvement of vocabulary, knowledge of exercises, self-confidence, and motivation		Of the 129 survey participants, 32 (24.8%) reported that Oneself increased their knowledge about back pain. Successful testimonials indicated that self-management was encouraged. No *P* values stated.	
	Hagerman et al (2017) [50]	Observational study			DAs^j^ for patients with low-risk PCa^k^		What are the informational needs of patients with low-risk PCa, and what are the essential aspects of treatment DAs that increase the likelihood of physicians recommending them to the patient?		Semistructured interviews found that “Physicians highlighted the importance of patient education and described the characteristics of a low-risk PCa DA that would increase the likelihood of its use in clinical practice.” Encourage patients to take their time in decision-making. Frankly inform on posttreatment side effects. Incorporate physician recommendations on content and mode of delivery. No *P* values stated.	
	Kim et al (2002) [51]	Observational study			Website comprising a situational approach to the organization of disease-specific patient information		Interface usability, personal relevance of retrieved information, comprehension of retrieved information.		Responses (n=37) yielded high ratings for the following: interface usability (4.6/5); personal relevance of information found (4.7/5); comprehension of information (4.8/5). No *P* values stated.	
	Meppelink et al (2015) [52]	Observational study			Colorectal cancer screening messages divided into high-literacy and low-literacy groups, with and without illustrations		Information recall, attitudes, intention to undergo screening		Spoken messages about colorectal cancer screening improved recall (*P*=.03) and attitudes (*P*=.02) compared with written messages in individuals with lower health literacy. Animations alone failed to improve recall, but when combined with spoken text, they significantly improved recall (*P*=.02).	
	Mayer et al (2003) [53]	Literature review			A theory designed to format multimedia content to optimize patient education		Overloading, speed of content delivery, and misalignment of textual and visual cues		Narration has better transfer of information than on-screen text. Learner-controlled segments increase transfer of information. Graphics and corresponding text should be aligned visually. Signals also improve transfer. No *P* values stated.	
**Stage of patient journey**										
	Biernatzki et al (2018) [54]	Cross-sectional descriptive			Evaluation of the informational needs of patients with diabetes		Treatment process, course of disease, abnormalities of glucose metabolism, and diabetes through the life cycle		Information needs among patients with diabetes is poorly investigated, although in high demand. No *P* values stated.	
	Boyde et al (2009) [55]	Observational study			An investigation of the learning style and learning needs of patients with HF^l^		Questionnaire identifying preferred learning modalities		In total, 64% (55/86) of the participants reported a preference for multimodal learning style; 18% (15/86) preferred textual information; 11% (9/86) preferred auditory; and 7% (6/86) preferred kinesthetic. Signs and symptoms were ranked as the most important topics to learn about. No *P* values stated.	
	Hagenhoff et al (1994) [56]	Systematic review			Evaluation of the perceptions of both patients and nurses on the importance of educational content for patients with congestive HF		Questionnaire evaluating the importance of the following categories: anatomy and physiology; psychology; risk factors; medications, diet, and activity; and other		Patients and nurses rated all information as moderately to very important to learn. Patients often rated information as more important than nurses did. No *P* values stated.	
	Wehby et al (1999) [57]	Descriptive comparative study			Perceptions of RNs^m^ and patients concerning educational content on HF were analyzed		Ranking of categories of HF education in order of importance by patients and RNs		“Patients perceived the subscales of general HF information, risk factors, medications, prognosis, and signs and symptoms as more important to learn than the RNs (*P*<.05).” “Patients perceived diet information as less important to learn than the RNs (*P*<.05).” “Patients perceived all eight subscales as more realistic to learn than the RNs (*P*<.05). Although not in identical order, both groups ranked education related to medication and signs and symptoms as the 2 priority areas.” “Diet information was ranked eighth by the patients and third by the RNs.” No *P* values stated.	
	Clark et al (2004) [58]	Descriptive correlational study			Examination of perceived learning needs of patients with heart failure after discharge		“The Outpatient Heart Failure Learning Needs Inventory was used to rate the participants’ perceptions of the importance of educational topics on a 5-point Likert scale.”		“The findings indicated that the subjects perceived signs and symptoms and medications as most important to learn and diet, activity, and psychological factors as least important to learn. These findings are consistent with previous research and provide a framework on which to base the development of educational programs for patients with heart failure. A significant finding was that nearly 25% of the screened patients were unable to participate because they were unaware that they had been diagnosed with heart failure.” No *P* values stated.	
	Kiliç B et al (2015) [59]	Descriptive comparative study			Qualitative analysis of questionnaires examining perceptions of RNs and patients concerning educational content on HF were analyzed.		Themes related to the educational needs of patients about use of drugs. Themes related to lifestyle changes. Themes about the educational needs of the patients related to the characteristics of the disease ranking of categories of HF education in order of importance by patients and RNs.		“In this study, HF patients stated that they mainly need information about the effects and purposes of the drugs they used. The need for information about the management of the symptoms that affect daily activities are considered 2nd and the educational needs about the disease itself are considered 3rd in importance.” No *P* values stated.	
	Solomon et al (2018) [60]	Qualitative study			To build an evidence-based web-based patient information resource for patients with HIV		Transcribed interviews of stakeholders underwent qualitative content analysis		The interviewees suggested that descriptions of all members of the health care team involved with HIV care be included on the website. It was also suggested to organize the menu into health challenge categories for ease of navigation. No *P* values stated.	
	Liu et al (2017) [61]	Umbrella review			Aimed to identify the current evidence on health education–related interventions for patients with acute coronary syndrome or type 2 diabetes		Clinical outcomes, behavioral outcomes, psychosocial outcomes, and medical service use		Nurses and multidisciplinary teams were the most frequent health care professionals to provide education, and most educational interventions were delivered after discharge. Face-to-face sessions were the most common delivery formats of the patient educational interventions. The psychoeducational interventions were found to be effective in reducing smoking and admissions for patients with acute coronary syndrome. No *P* values stated.	
**Credibility and completeness**										
	Boyer et al (1998) [62]			Review		Review of *HONcode*^n^, a guideline designed to raise the quality of web-based patient education data		Guidelines to information providers, with the aim of raising the quality of web-based data available and helping to identify websites that are maintained by qualified people and contain reliable data.		The HONcode mainly includes the following ethical aspects: the author’s credentials, the date of the last modification with respect to clinical documents, confidentiality of data, source data reference, funding, and the advertising policy. No *P* values stated.
	Priyanka et al (2018) [63]	Observational study			Evaluation and analysis of web-based content pertaining to esophageal duodenoscopy for patients		GQS, Health on Net, Flesch-Kincaid Reading Ease, and Flesch-Kincaid Grade Level		Three websites were found to have high-quality, comprehensive, and authentic information: Healthline, Uptodate, and Emedicine. In total, 13 sites yielded moderate quality of information. The mean Flesch-Kincaid Reading Ease score was 46.92. The mean Flesch-Kincaid Grade Level was 11th grade. No *P* values stated.	
	Couper et al (2010) [64]	Cross-sectional survey			Analysis of the perceived importance of sources of health information on the web		Ranking of sources in terms of reliability and influence; use of the internet in age groups.		Internet use was more common at younger ages, increasing from 14% among those aged 70 years or older to 38% for those aged 40-49 years. Internet users rated health care providers as the most influential source of information for medical decisions, followed by the internet, family and friends, and media. No *P* values stated.	
	Volk et al (2013) [65]	Cross-sectional survey			Evaluation of ongoing studies regarding what the standards for DAs for patients should be		Voting system to develop criteria for DA standards		The review comprised 13 manuscripts on topics including current frameworks used to create health care content, health literacy, and the role of patient stories. No *P* values stated.	

^a^SMOG: Simple Measure of Gobbledygook.

^b^PEMAT: Patient Education Materials Assessment Tool.

^c^TRAK: Taxonomy for the Rehabilitation of Knee Conditions.

^d^VaPE: Video-Assisted Patient Education.

^e^JAMA: Journal of the American Medical Association.

^f^GQS: Global Quality Scores.

^g^SGSS: Sleeve Gastrectomy Scoring System.

^h^WAI: Web Accessibility Initiative.

^i^mHealth: mobile health.

^j^DA: decision aid.

^k^PCa: prostate cancer.

^l^HF: heart failure.

^m^RN: Regional nurse.

^n^HONcode: Health on the Net Foundation Code of Conduct.

#### Textual Information

A study by Ernest et al [12] used the *DISCERN* Tool to evaluate the quality of written information regarding hormone treatment and surgery, which was found to be poor [66]. DISCERN is a validated tool developed by an expert panel through a process of panel debate and health care information analysis. It is noted to be the “first standardised index of quality of consumer health information” [66]. The DISCERN Tool comprises 15 key questions that investigate publication reliability and details of treatment choices, followed by overall judgment of quality. Each question is answered on a 5-point scale ranging from no to yes.

The study by Vivenkanatham et al [18] evaluated textual information on polymyalgia rheumatica. The study concluded that for web-based health care content to be effective, readability must be accessible to people of all literacy levels. Similar conclusions on readability were also reached in the study by Maciolek et al [20], which reported that patient education materials are most effective when simple language accessible to a wide patient population is used. A quality assessment study of web-based content on rheumatoid arthritis analyzed the readability, applicability, and accessibility of patient education websites [21]. The mean reading level was found to be 12.1 (SD 2.3), according to the Flesch-Kincaid Readability Tool. This tool gives a measure of how difficult a passage written in English is to understand through analyzing factors such as word length, sentence length, and total number of syllables. It provides a grade level according to the US educational system ranging from fifth grade to college graduate [67]. The same study reported that 78% of the websites assessed were easy to navigate [21]. Importantly, only 33% of the websites were assessed to be user friendly for people who are visually or hearing impaired.

An evaluation of web-based information on disorders of sex development noted strengths, including the tendency of webpages to present focused information in chunks and in a logical sequence [12]. A semistructured interview study of general practitioners conducted by Hjelmager et al [13] revealed that health information technology apps require textual information that is targeted to patients and written with the input of health care professionals.

The study by Rofaiel et al [14] assessed the quality of website information about inflammatory bowel disease using the DISCERN score to assess reliability and the relevance of pertinent details. The mean DISCERN score for patient-searched websites was not statistically different from that for physician-recommended websites (3.21 vs 3.62, respectively; *P*=.16). Numerous studies [12,13,15-17] identified that readability, as analyzed by the Flesch-Kincaid Tool, of web-based information exceeded the recommended sixth to eighth grade reading levels [68].

In all, 3 studies evaluated the understandability and actionability of web-based patient information by using the validated Patient Education Materials Assessment Tool [12,17,20]. The tool comprises inventories (one for print and another for audiovisual content) that list desirable and undesirable characteristics of information and produce a numeric value for understandability as well as actionability (ie, how easy it is to act on given information). The scores range from 0% to 100%, and a higher score indicates that the text is more understandable or actionable. Web-based educational content pertaining to disorders of sexual development and tympanostomy tube placement yielded low actionability scores (mean scores 23% and 44.6%, respectively). Interestingly, a study by Maciolek et al [20] found that the Patient Education Materials Assessment Tool understandability score for academic institution patient educational material exceeded that for content created by private institutions (*P*=.02) and content from websites unaffiliated with a urologist (*P*=.01).

#### Discussion Boards or Web-Based Groups

The study by Cedars et al [27] reported on a study of patients with urethral strictures who participate in web-based discussions and share experiences to gain emotional support and find answers. Patients participating in these web-based groups were more often than not satisfied with their postoperative outcomes. These findings are supported by the study by Teaford et al [28], which explored the experiences of new mothers using a web-based postpartum forum. The study found that web-based forums provided a sense of community and a platform for sharing information. They identified five themes pertaining to the participants’ concept of a web-based community: social support, anonymity, in-groups, drama, and entertainment.

However, the findings of a study by Castaneda et al [29] highlight the importance of exercising caution with web-based forums. The study evaluated the content of peripheral arterial disease eHealth forums and found that 15.1% of the medical advice given on such platforms was inconsistent with guidelines. Furthermore, the study found that 10 of the 18 posts related to negative personal experiences with health care providers.

It is important to note that there may be differences in patient engagement when comparing groups that are run by health care (or affiliated) professional service providers with those set up by patients or lay individuals themselves. A study comparing the difference between the effects of peer-led and moderated groups found that moderated groups were often more active and therefore had higher patient engagement.

#### Video-Based Content

The study by Pedersen et al [31] found that 4 participants who were interviewed after watching a 14-minute educational video on whiplash injuries felt reassured, particularly because the video aligned with information that they had received at the hospital. The study by Finnegan et al [32] reported that people who visited an information hub where videos were embedded in the webpage spent longer than 2 minutes on that webpage, indicating that visitors with average reading capability watch and listen to the video while browsing the text. The study also reported that this information hub’s YouTube channel, which featured videos explaining the concept of herd immunity, was particularly successful, with visitors spending more than 6 minutes on that page [32].

In the context of managing health conditions, the study by Button et al [33] found that video-based content was particularly helpful for patients having physiotherapy for knee injuries because they were able to visualize the correct technique. Patient understanding and confidence were found to be improved by this intervention. In a separate study of 80 anesthetists using a Video-Assisted Patient Education intervention, 96% reported that patients felt that they had a better understanding of the information provided through the Video-Assisted Patient Education intervention, and 97.5% of the anesthetists felt that it was a useful form of patient education [34]. However, 46% noted increased anxiety caused by the intervention [34].

Interestingly, the study by Ferhatoglu et al [36] found in an evaluation of the quality of YouTube content on sleeve gastrectomy that patient experience and advertisement videos were significantly more popular than academic videos created by medical professionals, according to the Video Power Index (*P*<.001 and *P*<.003, respectively). The Video Power Index assesses video performance by comparing the video with leaders in its respective industry, measures its impact and effectiveness across all platforms, and facilitates strategies to cater to target audiences [69]. The study by Erdem and Sisik [37] reported similar findings and found no significant association between video traction (*likes*, *dislikes*, or *views*) and usefulness of the content (Spearman rank correlation ρ=−0.038, *P*=.61; ρ=−0.003, *P*=.97; and ρ=−0.118, *P*=.12, respectively). Interestingly, the study found no significant correlation in usefulness to video length (ρ=−0.106; *P*=.16) in contrast to the findings of the study by Biggs et al [38], which concluded that medical videos categorized as useful had a mean length of 6 minutes and 51 seconds, with the videos rated in the top 10 having a mean length of 14 minutes and 47 seconds.

Similar to previous findings, a study conducted to assess the quality of YouTube videos on cataract surgery concluded that videos created by medical organizations such as the National Health Service were significantly more useful in terms of educating patients about the procedure than videos sourced by independent medical professionals and other sources (*P*<.001) [30]. The study by Bademci et al [40] similarly concluded that medical topic videos on deep vein thrombosis sourced from medical professionals and hospitals were significantly more useful than videos from other sources (*P*<.001).

In a study evaluating YouTube content on varicose veins, it was found that most of the videos were dominated by the private health care sector and that the video content presented a distorted view of treatment options, consequently leading to a skewed patient perception of the therapeutic options available to them [39]. Not only were 32% of these videos found to be of poor quality, but videos from private medical companies were also significantly more likely to favor minimally invasive surgery over ligation and stripping than videos from other sources. Once again, there was no significant association found between quality of content and viewing frequency.

#### Visuals or Pictographs

Pictographs are pictorial representations of words or phrases. The study by Christensen et al [41] described the value of pictographs in health communication. The study presents the results of building and testing the game *Doodle Health*, which is designed to produce pictographs through crowdsourcing. It found that this method of pictograph creation yielded positive feedback from focus groups with regard to usability and comprehension. Analysis of the feedback found that 62.2% of the participants praised the game, with a respondent describing it as “engaging and easy to use.” However, the study also found that people from diverse cultural backgrounds have different styles of communication, which may render visually presented information ineffective for minority groups. Crowdsourcing pictographs was suggested as a potential solution to this concern.

#### Device Accessibility

A study by Gogovor et al [42] concluded that the next generation of web-based educational health care programs should integrate apps for reasons of (1) accessibility, (2) flexibility, and (3) security and trustworthiness. Almost all the studies in our review used web-based platforms and required devices that accessed the web, with some studies requiring devices that accessed the video-sharing platform, YouTube, specifically.

A study by Lüchtenberg et al [43] found that only 18% (25/139) of the health information websites evaluated achieved a high standard of accessibility for users who are visually impaired as assessed by the Web Accessibility Initiative guidelines. The study concluded that web-based educational health care content should at least meet the requirements of priority 1 (level A) and preferably priority 2 (level AA) of the Web Accessibility Initiative guidelines. Developers can ensure accessibility by, for instance, having text alternatives for all nontext content and using high contrast ratios of text and images [36].

Few studies used smartphones exclusively to deliver information. Describing the benefits of using a smartphone, the study by Bashi et al [44] noted that smartphone adoption is becoming widespread, meaning more people can be reached; however, studies on how apps should deliver information in terms of interface and content is particularly lacking. The study by Noel et al [45] assessed the impact of a mobile medical app on plastic surgery patient care. A total of 60 patients answered questionnaires pertaining to the app, and the results supported the app’s utility from the patient’s perspective. The mean score for the app’s ability to answer patient questions was 4.1/5, and 20% of the patients were prevented from unnecessarily calling the emergency department. The use of a smartphone also enabled patients to access information from different locations and allowed for real-time disease management. A systematic review by Nicholas et al [46] revealed that of the 11 studies of apps providing patient information, only a third covered core psychoeducational principles. Furthermore, the average user ratings were not correlated with either comprehensiveness or adherence to best practice guidelines (ρ=−0.11, *P*=.80 and ρ=−0.02, *P*=.96, respectively).

#### Stage of Patient Journey

A qualitative study by Gogovor et al [42] using health care professionals and patient focus groups found that health care professionals suggested stratifying access to information according to *stage of advancement* in terms of their chronic pain management. A qualitative study of *Oneself*, a website designed for use by patients with chronic lower back pain, found it to be an effective adjunct to doctor-patient consultations because patients found that they were unable to have all their queries answered in the limited time of a consultation [49].

At different stages of a patient journey, decision aids may be used as adjunctive decision-making tools to support patients. In the study by Hagerman et al [50], the authors conducted semistructured interviews of 33 physicians to identify the desirable characteristics of decision aids. Of the 33 physicians, 20 (61%) stated that patients should be educated on the lack of urgency with regard to making a treatment decision. Of the 33 physicians, 28 (85%) agreed that decision aids should be provided to the patient after the consultation when the patient is at home. Furthermore, 36% (12/33) of the physicians deemed it *very* or *extremely* important that decision aids are designed to be used during and after consultations.

Tailoring information delivery to the stage of the patient journey is further supported by the results of the study by Kim et al [51] who developed a system to organize web-based disease-specific information according to a situational knowledge base model. The approach categorizes information about a specific disease (eg, thyroid cancer) into sections corresponding to discrete clinical events (eg, presentation, fine-needle aspiration biopsy, and diagnosis). In all, 75 patients completed a questionnaire evaluating the website, which found mean usability to be 4.6/5, personal relevance of received information 4.7/5, and comprehension of received information 4.8/5.

#### Credibility and Completeness of Information

A study by Jamison et al [47] found that 86% of the apps for pain conditions reported were created with no involvement by health care professionals. A comprehensive study by Bae et al [30], assessing the quality of the content of YouTube videos for cataract surgery patient education, found that there was an abundance of videos simply showing patients undergoing a live procedure. More than 20% of the educational videos were commercial and hence potentially misleading. This may make it challenging to find high-quality, comprehensive educational videos on the web.

A study by Pithadia et al [35] used the American Academy of Dermatology guidelines as a benchmark to evaluate the accuracy of patient information YouTube videos on psoriasis treatments. It concluded that 12% of the videos contained high-quality patient education content, and most of them were not patient-centric. Similarly, the study by Ferhatoglu et al [36] used the *Journal of American Medical Association* benchmark criteria to assess the educational quality of sleeve gastrectomy YouTube videos and found that this score was significantly higher in university-affiliated physician videos than in other videos (*P*<.001).

The Health on the Net Foundation Code of Conduct (HONcode) presents a set of eight principles designed to set the quality standard for web-based patient information [62] (Textbox 1). A study by Laversin et al [70] compared 165 HONcode-certified websites with 165 noncertified websites. Only 0.6% of the noncertified websites conformed to the principles of the HONcode compared with 89% of the certified sites (*P*<.10). As the study followed certified websites 6 months after certification, the effect of the HONcode certification shows short-term sustainability.

**Quality standard (adapted from the study by Laversin et al [70]).**

**Eight Principles Designed to Set the Quality Standard for Web-Based Patient Information**

1. Authoritative: qualifications of the authors indicated
2. Complementarity: information should support the doctor-patient relationship
3. Privacy: personal data collected by the site kept private
4. Attribution: cite all references
5. Justifiability: back up claims relating to benefits and performance
6. Transparency: accessible presentation, accurate email contact
7. Financial disclosure: identify funding sources
8. Advertising policy: clearly distinguish advertising from editorial content

## Discussion

### Principal Findings

**Figure 2:**
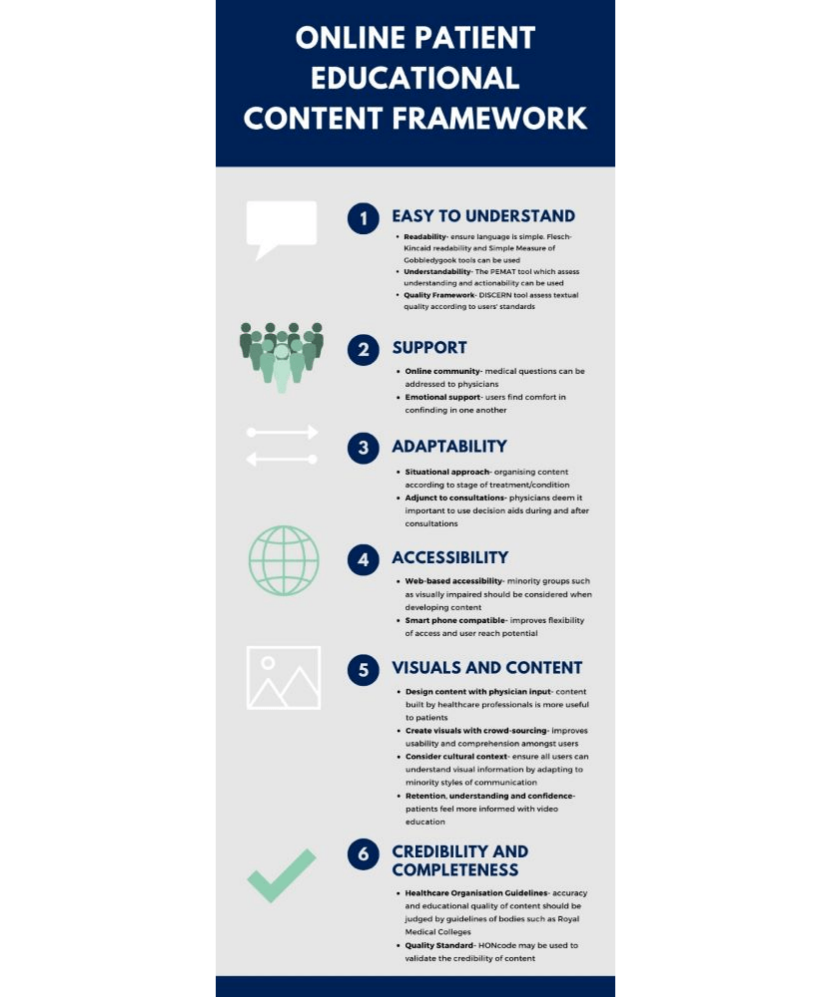
Infographic framework for modes of engagement for web-based health care content. HONcode: Health on the Net Foundation Code of Conduct; PEMAT: Patient Education Materials Assessment Tool.

In today’s world of access to knowledge often being initially web-based, it is of importance for health care professionals to be able to create effective content. This is further emphasized in the current environment where minimum contact between patients and health care providers is required. We performed a wide scoping review of the literature to identify the features of web-based content and other telemedicine requirements that may improve quality of engagement with web-based health care content in this growing field. Using these results, we have developed a framework (Figure 2) to facilitate the development of web-based patient health care content. All the categories except for one (visual or pictograph) were reported on by 3 or more papers. We included the visual or pictograph category after discussion because it was felt to be a relevant and important means of communicating information. Although the features identified are, on reflection, intuitive, the framework arms the content creator with the best available strategies in making the content engaging and hence effective.

Other similar frameworks such as the International Patient Decision Aid Standards (IPDAS) and the Standards for Universal Reporting of Patient Decision Aid Evaluation Studies (SUNDAE) checklists [65,71] have been developed for the evaluation of decision aids. However, to the best of our knowledge, this is the first framework to specifically focus on engagement with web-based content rather than a checklist approach to ensuring that decisions are made with appropriate consideration given to all relevant issues and options. A study design published by Knerr et al [72] aimed to evaluate patient behavior in response to a patient decision aid based on the IPDAS standards but has not reported results. Although patient decision aids can be a form of web-based content, efforts up to now have been directed toward ensuring transparency and trust in imparting information rather than ensuring the ability of web-based material to engage the user [73]. The need to engage people in health care content has been overlooked. We propose that this framework be used to improve engagement, which in turn will improve adherence with medical treatment and hence improve outcomes. Nevertheless, the rigorous process used by the IPDAS and SUNDAE developers is one that we would be interested in following in refining and adapting the framework arrived at through this literature review.

Further work will involve a co-design process with all stakeholders (including patients) to refine the insights we have gained from the studies regarding engagement with web-based content. The personalization of health care content may have bearing on the issue of engagement. Studies have shown that it is possible to *segment* the population according to the likelihood of responding to health care messages [74,75]. Although our framework provides the best evidence available relating to engagement with web-based content, the holy grail may lie in developing further the field of psychographics for health care. Although researchers have investigated the way that segmentation affects a defined intervention, the effect of segmentation itself is yet to be assessed in a meaningful manner [75]. The internet is able to bring together varied but related content using the concept of the semantic web and the application of folksonomies [76]. The confluence of Web 3.0 (to crowdsource content relevant to a desired health care behavior), psychographic segmentation (including segmentation based on the proposed framework), and machine learning may provide a way forward. We have developed a Web 3.0 health care content platform (Health Shared) and intend to use it for this purpose.

### Limitations

The findings of this review should be considered in the context of several limitations. The principal limitation is that most of the components of this suggested framework are not supported by strong evidence. The studies were heterogeneous in their aims, interventions, and outcomes, and some were of poor methodological quality. Few studies discussed the effect of the platform used on patient engagement—for example, smartphone app versus website versus commercial health care information—which may play a role in patient engagement. However, given that most of the studies discussed in the review were general scans of available websites, the framework developed is largely applicable to the website development platform. In addition, few studies describe the differences between informational sites and other modes-of-engagement systems that provide the ability for patient input and enable patients to contact their provider or providers; therefore, we were not able to compare these patient information platforms.

Furthermore, because only the PubMed database was searched, studies are likely to have been omitted from this review. Despite this limitation, the components of the framework are intuitive, and we believe that its application may be beneficial to health care providers and content creators. Evaluation and subsequent validation of the proposed framework by key stakeholders, including patients, clinicians, and content creators, would increase the robustness.

### Conclusions

There is a paucity of high-quality data relating to the factors that improve users’ quality of engagement with web-based health care content. Our framework summarizes the reported studies, which may be useful to health care content creators. Evaluation of the utility of web-based content to engage users is of significant importance and may be accessible through tools such as the Net Promoter score. Web 3.0 technology and development of the field of psychographics for health care offer further potential for development [75]. Future work may also involve improvement of the framework through a co-design process.

Although there are often specific health care issues needing to be addressed in response to crisis situations, we believe that this work is more generally important in facilitating patient activation and patient-supported self-management, which are two major pillars in how health care systems need to realign to keep up with increasing demand.

## Abbreviations

HONcode: Health on the Net Foundation Code of Conduct
IPDAS: International Patient Decision Aid Standards
SUNDAE: Standards for Universal Reporting of Patient Decision Aid Evaluation Studies
